# Digital Subtraction Phonocardiography (DSP) applied to the detection and characterization of heart murmurs

**DOI:** 10.1186/1475-925X-10-109

**Published:** 2011-12-20

**Authors:** Mohammad Ali Akbari, Kamran Hassani, John D Doyle, Mahdi Navidbakhsh, Maryam Sangargir, Kourosh Bajelani, Zahra Sadat Ahmadi

**Affiliations:** 1 Department of Biomechanics, Science and Research Branch, Islamic Azad University, Tehran, Iran; 2 Department of Anesthesiology, Cleveland Clinic Lerner College of Medicine, Case Western Reserve University, Ohio, USA; 3 Department of Mechanical Engineering, Iran University of Science and Technology, Tehran, Iran; 4 Department of Medical Physics & Biomedical Engineering, Tehran University of Medical Sciences, Tehran, Iran

**Keywords:** Digital subtraction, phonocardiography, MATLAB, Murmurgram

## Abstract

**Background:**

During the cardiac cycle, the heart normally produces repeatable physiological sounds. However, under pathologic conditions, such as with heart valve stenosis or a ventricular septal defect, blood flow turbulence leads to the production of additional sounds, called murmurs. Murmurs are random in nature, while the underlying heart sounds are not (being deterministic).

**Innovation**

We show that a new analytical technique, which we call Digital Subtraction Phonocardiography (DSP), can be used to separate the random murmur component of the phonocardiogram from the underlying deterministic heart sounds.

**Methods:**

We digitally recorded the phonocardiogram from the anterior chest wall in 60 infants and adults using a high-speed USB interface and the program Gold Wave http://www.goldwave.com. The recordings included individuals with cardiac structural disease as well as recordings from normal individuals and from individuals with innocent heart murmurs. Digital Subtraction Analysis of the signal was performed using a custom computer program called **Murmurgram**. In essence, this program subtracts the recorded sound from two adjacent cardiac cycles to produce a difference signal, herein called a "murmurgram". Other software used included Spectrogram (Version 16), GoldWave (Version 5.55) as well as custom MATLAB code.

**Results:**

Our preliminary data is presented as a series of eight cases. These cases show how advanced signal processing techniques can be used to separate heart sounds from murmurs. Note that these results are preliminary in that normal ranges for obtained test results have not yet been established.

**Conclusions:**

Cardiac murmurs can be separated from underlying deterministic heart sounds using DSP. DSP has the potential to become a reliable and economical new diagnostic approach to screening for structural heart disease. However, DSP must be further evaluated in a large series of patients with well-characterized pathology to determine its clinical potential.

## Background

In the healthy cardiovascular system, blood flow is generally laminar in character. Under certain pathologic conditions, such as a narrowing of a heart valve or a small hole in the ventricular septum, blood flow becomes turbulent, and can be heard as a noise known as a murmur [1]. One difficulty for clinicians is that this murmur is only part of the total sound signal emitted from the heart, which also contains underlying regular heart sounds. This fact necessarily complicates the listening process.

Listening to the emitted sounds from the heart using a stethoscope (auscultation) is a frequent first step in diagnosis. It is often followed by echocardiography when the auscultatory findings are abnormal. However, the lack of reliability of ordinary auscultation and the expense and awkwardness of echocardiography make it desirable to develop a more practical, inexpensive, reliable, non-invasive approach to auscultation, one that could also be adapted for continuous monitoring [2-6]. In order to better identify any pathology found and make as detailed as possible any diagnosis, it would also appear to be helpful if the murmurs could somehow be separated from the underlying deterministic heart sounds.

The present study seeks to apply a new (unpublished, unpatented) analytical technique, known as Digital Subtraction Phonocardiography (DSP), to develop a noninvasive means for the detection and characterization of heart murmurs, such as those resulting from heart valve lesions or other types of cardiac pathology. The proposed technique is fundamentally different from previous phonocardiographic signal processing efforts (Rangayyan and Lehner 1988 [7]; Khadra et al 1991 [8]; Bentley and McDonnell 1994 [9]; Durand et al 1993 [10]; Guo et al 1994 [11]; Durand and Pibarot 1995 [12]; Tranulis et al 2002 [13]).

The DSP technique is fundamentally different from those efforts that it starts by constructing a difference signal between two time-adjacent heart cycles, which we herein call a "murmurgram". Furthermore, based on a deterministic plus random component phonocardiogram model that seems quite plausible to us, we show through both mathematical reasoning and computer simulation how murmurgrams would be expected to behave. In addition, it is our empirical (although necessarily preliminary) observation that murmurgrams in patients with abnormalities like mitral regurgitation or aortic stenosis are different from normal controls.

## Methods

We collected the heart sounds at the Pediatric Clinic of Modares Hospital in Tehran between 2010 to 2011. Data were collected from a total of 59 cases, including 7 normal cases, with an age range of 5 to 26 years. Written informed consent was obtained from the patients for publication of the case reports and accompanying images. A copy of the written consent is available for review by the Editor-in-Chief of this journal. The patients (except for the normal controls) had a history of heart murmur, which included 22 with a ventricular septal defect (VSD), 7 with an atrial septal defect (ASD), 10 with Tetralogy of Fallot (TOF), 4 with aortic stenosis (AS), 5 with pulmonary stenosis (PS) and 4 having mitral regurgitation (MR). In all cases, the diagnosis was confirmed by echocardiography. The summary of medical history and diagnostic findings for some of the cases is presented in Table 1.

**Table 1:** Recorded heart sounds for some of the patients studied.

Patient Study Number	Patient age	Patient weight	Cardiac disease
Simulated Case1	....	....	Healthy

Simulated Case2	....	....	VSD

Simulated Case3	....	....	ASD

Case 1	23	78	Healthy

Case 2	26	69	Healthy

Case 3	8	22	VSD

Case 4	15	49	ASD

Case 5	6	16	TOF

Case 6	18	55	PS

Case 7	5	15	MR

Case 8	8	20	AS

### Recording Setup

**Figure 1:**
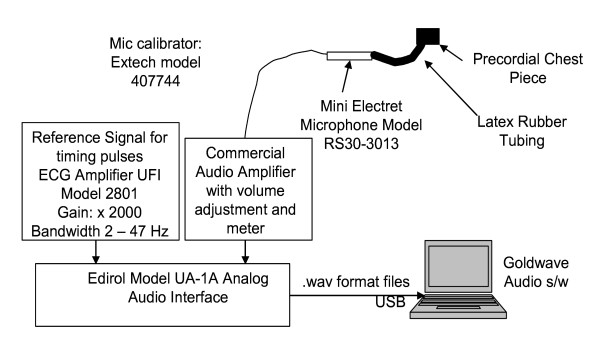
**The schematic of the system which has been used for collecting data ample**.

The data were recorded using a laptop computer-based phonocardiographic recording system developed at the Science and Research Branch of Islamic Azad University. Figure 1 shows the system. A miniature electret microphone, connected to a precordial chest piece, is connected to a commercial audio amplifier whose output is then digitized at 44 KHz with 16 bits resolution. A similar arrangement was also used to record the electrocardiogram (ECG), which was recorded to assist in the identification of the start of each cardiac cycle. (Because the ECG was digitized using a sound card that was high-pass filtered at around 20 Hz, this signal was somewhat different than those recorded under full-bandwidth conditions. Despite this, however, the QRS complex of the ECG can still be used as a marker of the beginning of each cardiac cycle.) This setup was placed on a mobile cart for easy recording in cardiology clinics and elsewhere. Table 2 shows the equipment which was used for recording the data.

**Table 2:** Equipment list which was used in this project.

	Equipment	Remark
**1**	Precordial Chest Piece	Hull Anesthesia

**2**	Latex Rubber Tubing	From stethoscope

**3**	Mini Electret Microphone	Realistic Model 3303013

**4**	Sound Level Calibrator, 94dB	Extech Model 407744

**5**	Commercial Audio Amplifier with volume adjustment and meter	

**6**	ECG Bioamplifer	UFI Model 2122i

**7**	USB Audio Interface	EDIROL Model UA-1EX

Microphone calibration was performed using a Extech Sound Level Calibrator Model 407744 which produces a sinusoidal wave at 1 KHz with 94 dB SPL intensity. By comparing any recorded sounds to the calibration recordings, it is then possible to obtain absolute sound intensity measurements. In order to achieve high-quality recordings, the clinical recording environment was kept completely silent, with the patients lying in the supine position. Each recording was divided into five sections of 3 minutes each. Care was taken to ensure that borborygmi sounds (from stomach and intestines) and other artifacts were not present.

All sounds were recorded using the Goldwave software (Version 5.55), which includes tools for recording, playing, filtering, and analyzing sounds. Using this software, we also deleted any electrical utility frequency (50 Hz) from the ECG recordings. Although GoldWave presents a wide range of digital filters, further filtering was done in MATLAB including Cancellation DC drift and Low & High Pass Filtering. The detailed code can be seen in the additional file 1. As we generate the murmurgram by subtracting two consecutive cycles, the only issue in children in how to find the exact location of the R-peaks. Pan-Tompkins algorithm was a good choice and great sampling rate of the recording system was helpful in this regard.

Furthermore, The rate of heart beats has not any influence on the sound signals from our point of view and based on the experiments which were done by us. The detail method of recording the data can be found in reference [6].

### Technical and Analytical Issues

**Figure 2:**
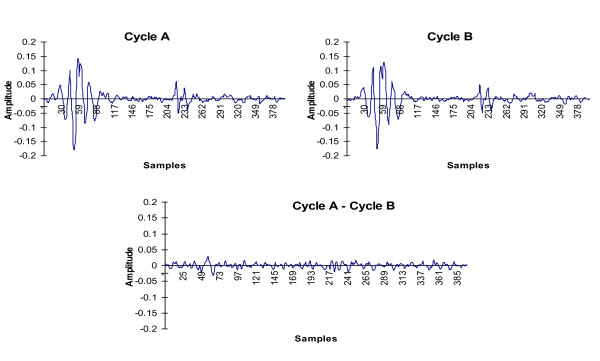
**The schematic of result of subtracting two successive heartbeats to construct a "murmurgram"**.

We used MATLAB in conjunction with the signal processing and real-time data acquisition toolboxes as the computational heart of the system. Two time-adjacent phonocardiogram cycles, PCG-1 and PCG-2, are obtained using the QRS complex of the ECG as a marker for the start of each cardiac cycle. The difference of the two PCG cycles is a *murmurgram*. Figure 2 illustrates the PCG subtraction method.

Note that this process is complicated by the fact that, due to the physiological variability, not all cardiac cycles are of the same length. Consequently, prior to data analysis and the construction of the murmurgrams, any data collection must first be subjected to a preliminary analysis to determine the longest duration cardiac cycle present. A raw murmurgram for a legal cycle i is then formed as the difference between PCG (cycle i) and PCG (cycle i +1).

**Figure 3:**
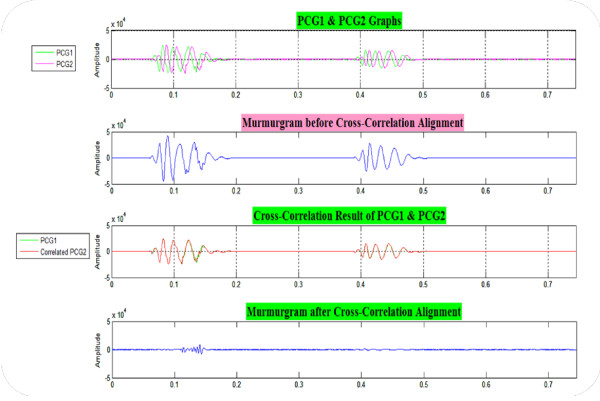
**The cross-correlation alignment and subtraction of two successive heartbeats to construct a "murmurgram"**. PCG1 and PCG2 are two successive phonocardiogram cycles.

As noted earlier, natural variations in the time between the QRS complex and the onset of the first heart sound (likely largely as a result of respiration) necessitates the use of an alignment procedure based on the cross-correlation alignment between any two PCG cycles being subtracted to form a murmurgram. Figure 3 shows the cross-correlation alignment and subtraction steps.

Following the murmurgram construction, we apply color spectrographic analysis to supplement the time-domain murmurgram. Signal intensity to color is mapped as follows: Red > Orange > Yellow > Green > Blue > Black. These color spectrograms use colors to denote signal intensity at a particular time and frequency. Our preliminary observations suggest that a normal murmurgram is fairly "flat" (uniform in character) and "low in intensity" in both the time and frequency domains, while this is expected not to be the case for patients with heart murmurs. (We expect that once a large corpus of cases have been collected that we will be able to replace vague concepts such as "flat" and "low in intensity" with something far more specific).

Finally, it is often important to be able to characterize murmurs in terms of their distribution over the cardiac cycle; e.g., systolic or diastolic. For instance, almost all murmurs that occur during diastole are abnormal.

## Results

### Simulated Case 1

**Figure 4:**
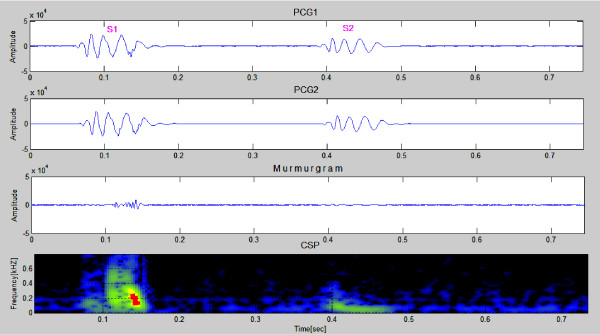
**Simulated normal PCGs (first & second panels)**. The murmurgram and corresponding color spectrogram are shown in the third and bottom panels. (Simulated Case 1).

Figure 4 shows samples of simulated PCGs (first & second panels) from a simulated healthy heart. The murmurgram and the corresponding color spectrogram are shown in the bottom two panels. Each simulated PCG is of 1 cardiac cycle covering an elapsed time of 0.7 second. Note that murmurgram is "flat" and has frequency components largely under 200 Hz.

### Simulated Case 2

**Figure 5:**
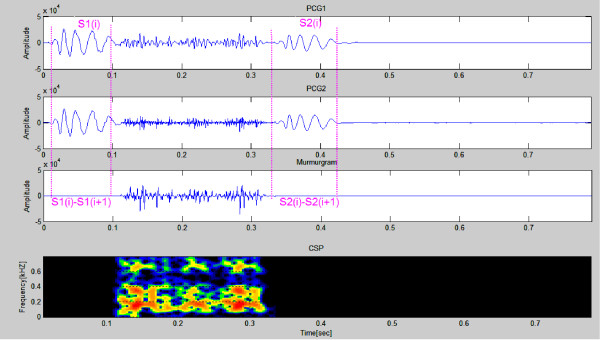
**Simulated PCGs for a patient with a VSD (first & second panels)**. The murmur can be seen to have frequency components that extend to around 700 Hz (Simulated Case 2).

Two successive heartbeats of data for a simulated VSD case are shown in the first and second panels in Figure 5. The murmurgram (third panel) between S1and S2 isn't "flat". The spectrographic graph, shown at the bottom of the figure, indicates that the murmur has frequency components extending to 700 Hz.

### Simulated Case 3

**Figure 6:**
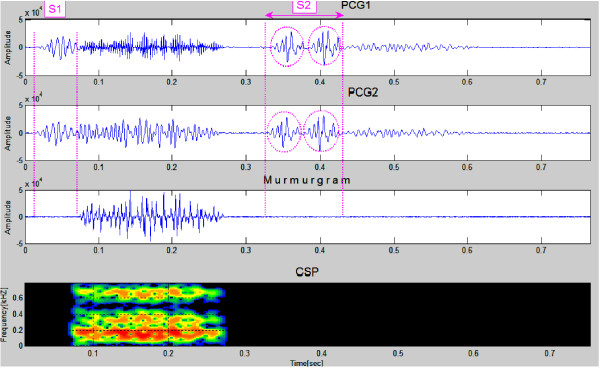
**Sample PCGs (first & second panels) from a simulated ASD patient**. Murmurgram (third graph) between S1 and S2 isn't "flat". The murmur has frequency components that extend to over 600 Hz. The second heart sound is clearly split in both PCG tracings (Simulated Case 3).

Figure 6 shows PCGs from a simulated ASD case. The murmurgram and corresponding color spectrogram are shown in the bottom two panels. The simulated PCG is of 1 cardiac cycle covering an elapsed time of 0.7 seconds. Note that the murmurgram isn't "flat" between S1and S2. The mid systolic murmur in the murmurgram in this case has frequency components that extend to around 600 Hz.

### Clinical Cases 1 and 2

**Figure 7:**
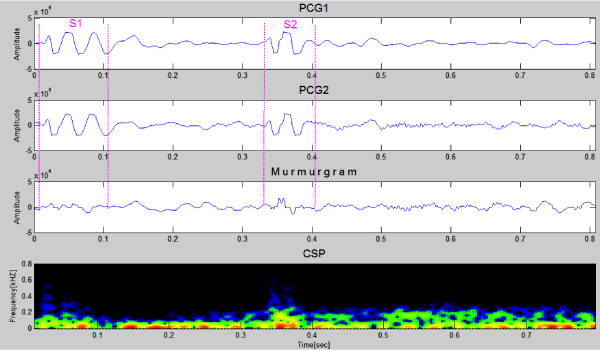
**This image shows PCG samples (first & second graphs) from a healthy 23 year old girl with an innocent heart murmur**. The murmurgram and the corresponding color spectrogram are shown in the third and fourth panels. The murmurgram in this case is relatively "flat", and the frequency content of the murmurs is largely under 150 Hz (Clinical Case 1).

**Figure 8:**
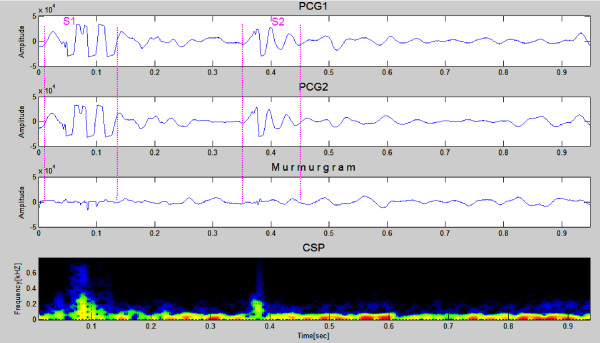
**This shows PCG samples (first & second panels) from a healthy 26 year old man with an innocent heart murmur nother sample figure title**. The murmurgram and the corresponding color spectrogram are shown in panels three and four. The murmurgram is relatively "flat" and the frequency of the murmur is largely under 150 Hz (Clinical Case 2).

Figures 7 and 8 show PCG samples (first & second panels) from a healthy 23 year old girl and a healthy 26 years old man, both with innocent heart murmurs. The murmurgrams and the corresponding color spectrograms are shown in the third and fourth panels in both figures. In Figure 7 the PCGs are of 1 cardiac cycle covering an elapsed time of 0.8 second while in Figure 8 the PCGs are of 1 cardiac cycle covering an elapsed time of 0.9 second. Note that in both figures the murmurgram is flat and the frequency of the murmurs is largely under 150 Hz.

### Clinical Case 3

**Figure 9:**
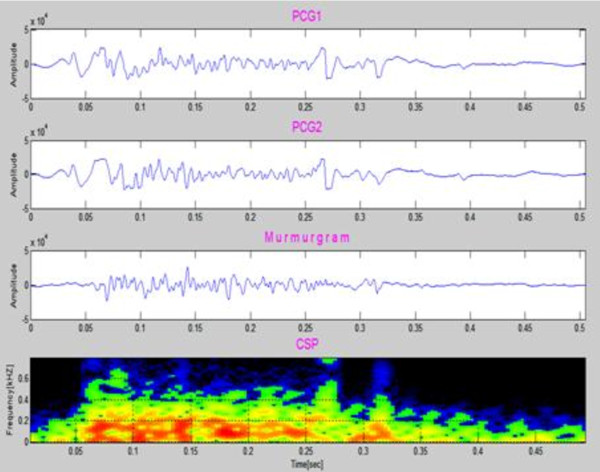
**Sample PCGs (first & second panels) from a patient with a VSD**. Note that the murmurgram (third graph) between S1 and S2 isn't "flat" (uniform in character) in behavior. The murmur has frequency components that extend to around 600 Hz (Clinical Case 3).

**Figure 10:**
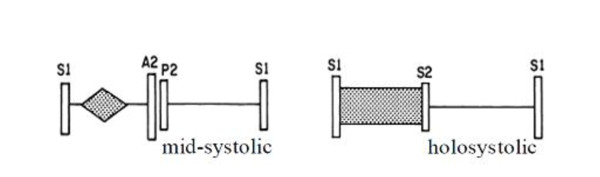
**A schematic diagram comparing mid systolic and holosystolic murmurs**.

The Figure 9 presents PCG samples (first & second panels) for a case of VSD in an 7 year old girl. 1 cardiac cycle is shown. The murmurgram (third panel) between S1 and S2 is not "flat". The spectrographic graph, bottom of the figure, indicates that the murmur has frequency components extending to 600 Hz. Referring to the murmurgram, we see that the position of the murmur is between S1 and S2 and that it is a holosystolic murmur as shown in Figure 10.

### Clinical Case 4

**Figure 11:**
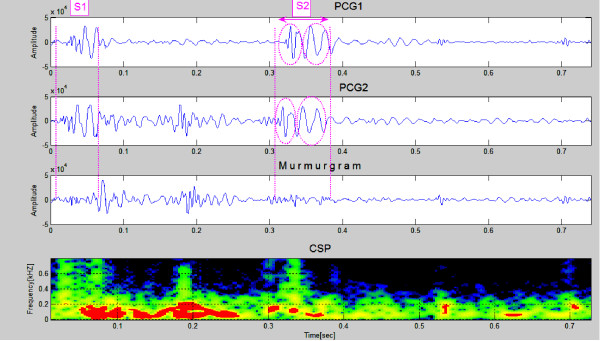
**Sample PCGs (first & second panels) from a patient with an ASD**. The murmurgram (third panel) between S1 and S2 isn't "flat". The murmur has frequency components that extend to around 400 Hz. The second heart sound is clearly split in both PCG waveforms (Clinical Case 4).

Two successive heartbeats of data for an 15 year old boy with an ASD are shown in Figure 11. Note that the murmurgram is not "flat" between S1 and S2. The murmur has frequency components that extend to around 400 Hz. The physiological explanation for ASD acoustic behavior is as follows: The murmur is characteristically mostly mid systolic, and occurs because an increase in blood leaving from the right ventricle in turn causes an increase of the load presented to the right ventricle, which in turn makes the duration of the systole longer. This is associated with a splitting of S2 (see red circles).

### Clinical Case 5

**Figure 12:**
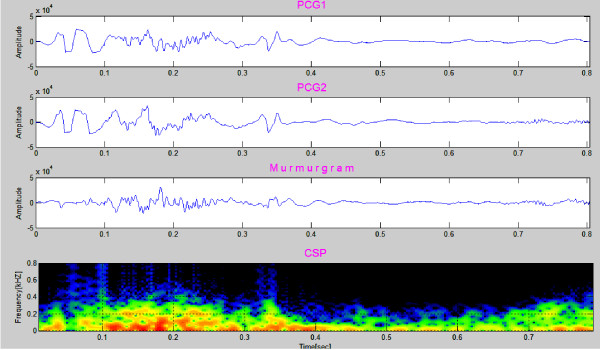
**A recording from a Tetralogy of Fallot 8 year old patient**. The murmurgram shows a systolic murmur and with frequency components extending to around 400 Hz (Clinical Case 5).

Data for an 8 years old boy with TOF are presented in Figure 12, The murmurgram (third panel) between S1 and S2 is not "flat", and corresponds to a systolic murmur with frequency components extending to around 400 Hz. Note that in the case of TOF (VSD, right ventricular hypertrophy, PS, and overriding aorta), both VSD and PS have systolic murmurs. The result is a systolic murmur resulting from two sources (Figure 12).

### Clinical Case 6

**Figure 13:**
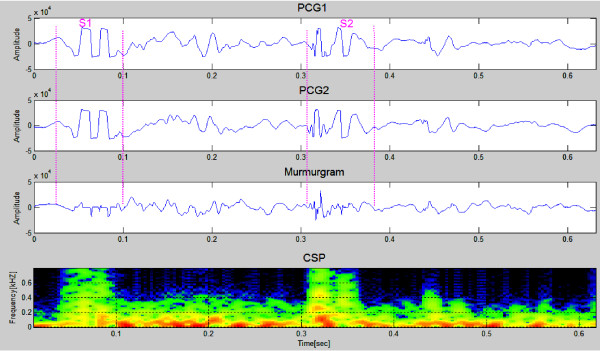
**Pulmonary stenosis in a 18 years old patient**. The murmur is systolic in timing and frequency components extend to over 500 Hz (Clinical Case 6).

Data for a 18 years old boy with PS are presented in Figure 13. The murmurgram (third graph) between S1and S2 is not "flat". This systolic murmur has frequency components to over 500 Hz. In Pulmonary Stenosis (PS), the blood is not able to easily enter into the pulmonary artery. This condition is characterized by a harsh systolic murmur (Figure 13).

### Clinical Case 7

**Figure 14:**
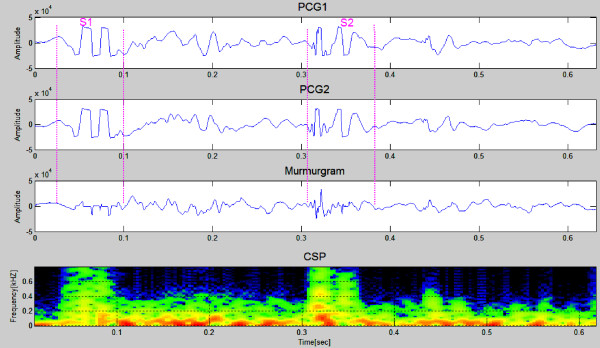
**Mitral regurgitation in a 5 year old patient**. The murmurgram shows a systolic murmur with frequency components extending to 400 Hz (Clinical Case 7).

Figure 14 is from a 5 year old girl with MR. The murmurgram (third panel) between S1 and S2 is not "flat". A systolic murmur with frequency components extending to 400 Hz is evident. Note the presence of a holosystolic murmur with an apparent early diastolic component (likely due to rapid antegrade flow through the mitral orifice).

### Clinical Case 8

**Figure 15:**
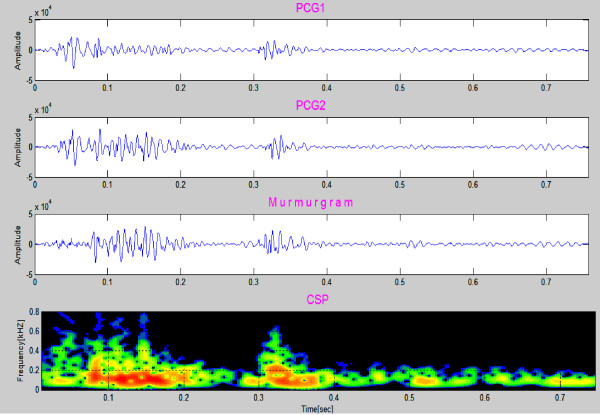
**Aortic stenosis in a 8 year old patient**. The murmur is between S1 and S2 and in this region the murmurgram is not "flat", with frequency components extending to about 400 Hz (Clinical Case 8).

Figure 15 presents data for an 8 year old boy with AS. The recording consists of 1 heart cycle and takes 0.7 second. The murmur is between S1 and S2 and the murmurgram (third graph) between S1 and S2 is not "flat". Frequency extends to about 400 Hz. Aortic stenosis (AS) is associated with a harsh mid systolic murmur in aorta area. In this case the murmur starts after S1 and reaches to maximum in the middle, 350~400 Hz frequency, and shrinks to a minimum before S2.

## Discussion

In this study ECG-synchronized digital subtraction and spectrographic analysis is used to study heart sounds and murmurs in an entirely new way. While older phonocardiograph designs remain useful to study cardiac disorders, this system offers two new dimensions to conventional graphical and auscultatory methods. These are: (1) the ability to separate the deterministic component of heart sounds from murmurs by digital subtraction; and (2) the ability to apply spectrographic analysis to the extracted murmur signals.

In this system we used digital signal processing techniques to construct a "murmurgram", defined as the resulting signal when one subtracts PCG cycle i+1 from PCG cycle i. A murmurgram is thus simply the difference between the acoustic emissions of two successive heart beats. In practice, the QRS complex of the ECG is used as a marker of the beginning of each cardiac cycle so that any two successive PCGs can be aligned and subtracted to produce a murmurgram. Also note that another series of murmurgrams could be obtained, for example, by subtracting PCG cycle i+2 from PCG cycle i and so on. For now we are concentrating on the use of "nearest neighbor" PCG cycles. Based on this model, a normal murmurgram should be more or less flat across the cardiac cycle (within the limits of system noise effects and biological variability) while increases in the murmurgram signal are expected to occur in regions of the cardiac cycle associated with intra cardiac turbulent blood flow resulting from cardiac structural pathology.

Our approach is an improvement in the state of the art; it produces a murmur signal free of the underlying deterministic heart sounds. By isolating the cardiac murmur from the phonocardiogram in this manner, the system may allow physicians to detect and characterize cardiac murmurs that may be rather difficult to reliably detect by traditional means using a stethoscope, such as when the heart is beating quickly or when the heart sounds are faint.

## Conclusions

Cardiac murmurs can be separated from underlying deterministic heart sounds using Digital Subtraction Analysis. Digital Subtraction Phonocardiography has the potential to become a reliable and economical new diagnostic approach to screening for structural heart disease.

## Competing interests

The authors declare that they have no competing interests.

## Authors' contributions

MAA has done the sample recording along with KB and ZSA. MS has written the MATLAB code with MAA. KH and MN have coordinated the project, review the results and written the manuscript. JDD has revised the manuscript, reviewed the results, did the final data analyzing, and supervised the whole project. All authors read and have approved the final manuscript.

## Supplementary Material

Additional file 1 **MATLAB code**. Final alignment code for presenting the murmurs. Click here for file
