# Supplementary material for: Digital Subtraction Phonocardiography (DSP) applied to the detection and characterization of heart murmurs
Source: Biomed Eng Online. 2011 Dec 20;10:109. doi: 10.1186/1475-925X-10-109 (PMC3258229; doi:10.1186/1475-925X-10-109)
Supplement: Additional file 1 — MATLAB code. Final alignment code for presenting the murmurs. [file 1475-925X-10-109-S1.DOCX]

clc

clear all

close all

%**************************************************************************

% Loading the ECG file

%****For (.mat) Files****

fnam = input('Enter the Second PCG file name (exm: PCG2.mat) :','s');

load (fnam);

PCG2= wavedata(1,1:end);%this is the pcg signal.

fnam = input('Enter the First PCG file name (exm: PCG1.mat) :','s');

load (fnam);

PCG1= wavedata(1,1:end);%this is the pcg signal.

%**************************************************************************

%**************************************************************************

fs = 44100; % Sampling rate

a=length(PCG2);

b=length(PCG1);

N = min(a,b); % Signal length

t = [0:N-1]/fs; % time indexs1=PCG1;

PCG2=PCG2(1:N);

PCG1=PCG1(1:N);

%**************************************************************************

%Cross-Correlation

%**************************************************************************

X1=xcorr(PCG1,PCG2); %compute cross-correlation between vectors PCG1 and PCG2

[m,d]=max(X1); %find value and index of maximum value of cross-correlation amplitude

delay=d-max(length(PCG1),length(PCG2)) %shift index d, as length(X1)=2*N-1; where N is the length of the signals

%%%%%%%%%%%%%%%%%%%%%%%%%%%%%%%%%%%%%%%%%%%%%%%%%%%%%%%%%%%%%%%%%%%%%%%%%%%

if delay<=0 % Shift of signal "TO THE LEFT"

Sfifted_PCG2=[PCG2(1-delay:N) zeros(1,-delay)];%Sfifted_PCG2 is shift of PCG2 by delay

N1=min(length(PCG1),length(PCG2));

N2=min(N1,length(Sfifted_PCG2));

t2 = [0:N2-1]/fs;

s1=PCG1(1:N2);

s2=PCG2(1:N2);

s3=Sfifted_PCG2(1:N2);

raw_Murmurgram=s2-s1;

correlated_murmurgram=s3-s1;

end

%************************************************

if delay>0 % Shift of signal "TO THE Rigth"

Sfifted_PCG2=[zeros(1,delay) PCG2(1:N-delay)];%Sfifted_PCG2 is shift of PCG2 by delay

%Edited_Sfifted_PCG2=Sfifted_PCG2(delay:end);

N1=min(length(PCG1),length(PCG2));

N2=min(N1,length(Sfifted_PCG2));

N1=min(length(PCG1),length(PCG2));

N2=min(N1,length(Sfifted_PCG2));

t2 = [0:N2-1]/fs;

s1=PCG1(1:N2);

s2=PCG2(1:N2);

s3=Sfifted_PCG2(1:N2);

raw_Murmurgram=-(s2-s1);

correlated_murmurgram=-(s3-s1);

end

%********************************

% Plot PCGs

%********************************

figure(1)

ax(1)=subplot(311);

plot(t2,s1) %Plot signal s1

title('Two PCGs')

hold,plot(t2,s2,'r'); xlim([0 max(t2)]);ylim([-5 5]*10^4);;ylim([-5 5]*10^4);

grid on

legend('PCG1','PCG2',2);

ax(2)=subplot(312);

plot(t2,s1) %Plot signal s1

title('Correlation Result')

hold,plot([delay:length(s2)+delay-1]/fs,s2,'r'); xlim([0 max(t2)]);ylim([-5 5]*10^4); %Delay signal s2 by delay in order to align them

grid on

legend('PCG1','Correlated PCG2',2);

%^^^^^^^^^^^^^^^^^^^^^^^^^^^^^^^

ax(3)=subplot(313);

plot(t2,s1)

title('Correlation & Shift Result')%Plot signal s1

hold on

plot([delay:length(s2)+delay-1]/fs,s2,'r'); xlim([0 max(t2)]);ylim([-5 5]*10^4); %Delay signal s2 by delay in order to align them

hold on,plot(t2,s3,'g');hold off;

grid on

legend('PCG1','PCG2','Shifted PCG2',3);

linkaxes(ax,'x');%Synchronize limits of x vector in all subplots

%********************************

% Plot Murmurgrams

%********************************

figure(2)

subplot(311)

plot(t2,raw_Murmurgram); xlim([0 max(t2)]);ylim([-5 5]*10^4);

title(' Raw Murmurgram(Before Alignment)')

subplot(312)

plot(t2,correlated_murmurgram); xlim([0 max(t2)]);ylim([-5 5]*10^4);

title('Correlated Murmurgram (After Alignment)')
